# Phosphosite-specific co-regulation networks of MELK kinase: insights from integrative global phosphoproteomes

**DOI:** 10.1016/j.jgeb.2026.100705

**Published:** 2026-05-08

**Authors:** Noreen A Khan, Amal Fahma, Althaf Mahin, Athira Perunelly Gopalakrishnan, Prathik Basthikoppa Shivamurthy, Samseera Ummar, Athira C. Rajeev, Rajesh Raju

**Affiliations:** Centre for Integrative Omics Data Science, Yenepoya (Deemed to be University), Mangalore, Karnataka 575018, India

**Keywords:** MELK kinase, Phosphoproteomics co-regulation networks oncogenic signaling

## Abstract

Maternal embryonic leucine zipper kinase (MELK) is a serine/threonine kinase frequently overexpressed in aggressive cancers, yet the precise mechanisms governing its activation and signaling specificity remain poorly understood. Here, we present the first phosphosite-resolved co-regulation atlas of MELK through integrative *meta*-analysis of 3,825 global human phosphoproteomics datasets. Three phosphosites-S356, S505, and S529-emerge as dominant regulatory nodes, exhibiting high detection frequency and distinct co-regulation patterns. S356 and S505 form a tightly coupled proliferative-mitotic axis controlled by convergent Mitogen-Activated Protein Kinase Kinase (MAPK), Ribosomal S6 Kinase (RSK), Calcium/Calmodulin-dependent protein Kinase (CaMK), Hippo-related, and spindle-checkpoint kinases (NIMA-related kinase 4 (NEK4), Threonine Tyrosine Kinase/Monopolar Spindle 1 Kinase (TTK/MPS1)), whereas S529 functions as a partially antagonistic stress- and polarity-responsive module. Marker of Proliferation Ki-67 (MKI67) phosphosites co-vary with all three MELK sites across virtually all proliferative contexts, establishing a direct mechanistic link between MELK activity and clinical proliferation markers. Extensive networks of co-regulated upstream kinases, phosphatases, binary interactors, and downstream substrates further reveal functional segregation: S356/S505 primarily drive cell-cycle progression and chromatin organization, while S529 integrates calcium, metabolic, and cytoskeletal polarity signals. Kaplan–Meier survival analysis across TCGA cohorts further revealed that high expression of MELK, MKI67, and the mitotic checkpoint kinase TTK consistently predicts poor overall and disease-free survival in lung adenocarcinoma and hepatocellular carcinoma, reinforcing the strong phosphodynamic coupling between MELK activity and clinical proliferation markers. By demonstrating that MELK signaling is orchestrated through modular, site-specific phosphorylation logic rather than total protein abundance, this work establishes a new paradigm for understanding and therapeutically targeting this enigmatic oncogenic kinase.

## 1 Introduction

Maternal Embryonic Leucine Zipper Kinase (MELK), also known as Protein Kinase PK38, MPK38, or pEg3-is a conserved serine/threonine kinase that regulates cell cycle progression, proliferation, and stress responses.^1^, ^2^, ^3^ First identified in 1990 by Paris in Xenopus oocytes and embryos, the human ortholog was cloned in 1997 by Heyer and colleagues from mouse embryonic cells, revealing strong homology to the Sucrose Non-Fermenting 1/AMP-activated Protein Kinase (SNF1/AMPK) family.^4^ MELK belongs to the AMPK-related kinase subfamily, characterized by a conserved catalytic domain and regulatory modules, alongside kinases such as NUAK family SNF1-like kinase 1 (ARK5), Brain-selective kinase (BRSK), Microtubule Affinity-Regulating Kinase (MARK), Qin-induced kinase (QIK), Novel (nua) kinase (NUAK), and Sucrose Non-fermenting AMPK-Related Kinase (SNARK).^5^, ^6^ Although MELK shares functional overlap with Ca^2+^/calmodulin-dependent kinases (CAMKs),^7^ its sequence and regulation firmly place it within the AMPK family.^8^

The MELK gene encodes a 651-amino-acid protein with a molecular weight of ∼ 74.5 kDa.^9^, ^10^ Structurally, MELK comprises an N-terminal catalytic kinase domain and a C-terminal regulatory region containing a leucine zipper, KA1 domain, and TP dipeptide-rich segment that together form an autoinhibitory module.^11^, ^12^ MELK localizes to both the nucleus and cytoplasm in a cell cycle-dependent manner and interacts with partners such as Receptor for Activated C Kinase (RACK1) to enable precise spatiotemporal signaling.^13^ MELK activation requires phosphorylation at Thr167 and Ser171 and is modulated by the intracellular redox environment and calcium levels. Full activity depends on reducing agents like dithiothreitol or reduced glutathione, whereas physiological Ca^2+^ concentrations inhibit the kinase.^12^ The isolated kinase domain is intrinsically hyperactive compared with the full-length protein, whose C-terminal TP-rich and KA1 regions impose autoinhibitory control that can be alleviated through context-dependent autophosphorylation.^6^

Under normal physiology, MELK activity is tightly regulated. In cancer, however, it is frequently overexpressed in breast, colorectal, glioblastoma, and prostate tumors, where it drives proliferation, therapy resistance, and poor prognosis.^14^, ^15^ The MELK inhibitor OTSSP167 has shown preclinical promise and entered early-phase trials, though some studies challenge MELK’s essentiality in cancer cells, pointing to context-specific roles or compensatory pathways.^16^ Nevertheless, MELK remains an attractive therapeutic target in tumors dependent on mitotic signaling and stress-adaptive survival.^17^

Despite growing insight into MELK biology, its phosphosite-level regulation- particularly in disease, remains underexplored. Resources like PhosphoSitePlus document MELK phosphosites and interactions but lack co-expression analyses from differential phosphoproteomics datasets. Given that phosphorylation likely varies across conditions and shapes signaling outcomes, we applied a phosphosite-specific co-expression framework to map MELK’s interaction network. We hypothesize that distinct phosphorylation patterns at key predominant sites may correlate with tumor phenotype, progression, and therapy resistance, and that phosphosite-resolved interaction maps will uncover novel regulators, biomarkers, and precision oncology opportunities.

## 2 Materials and methods

### 2.1 Screening and compilation of global phosphoproteomics data for MELK phosphosites

Protein phosphorylation represents a critical post-translational modification (PTM) that regulates a wide array of cellular functions, such as signal transduction, metabolic pathways, and cell-cycle control.^18^ Recent progress in mass spectrometry has transformed phosphoproteomics into a robust, high-throughput tool for large-scale, unbiased identification of phosphosites.^19^ To build an exhaustive inventory of MELK phosphosites reported in human studies, a systematic PubMed search (https://pubmed.ncbi.nlm.nih.gov/) was conducted with the query “phosphoproteomics” OR “phosphoproteome” NOT “Plant” NOT “Review,” thereby excluding plant-related studies and review articles and resulting in a refined set of global human phosphoproteomics datasets that document MELK phosphorylation.

Only Class I sites-those with a localization probability of ≥ 75% and an A-score (Ambiguity score) of ≥ 13- were included to guarantee reliable site localization. The datasets were divided into two main categories: (i) quantitative differential datasets that assessed phosphorylation changes between experimental/disease states and corresponding controls using statistical testing, and (ii) qualitative profiling datasets that simply reported phosphorylation events under specific conditions without paired comparisons. For quantitative datasets, differential regulation of phosphosites was defined based on fold-change thresholds reported in the original studies, with sites generally considered upregulated when fold change was ≥ 1.3 and downregulated when ≤ 0.76, subject to statistical significance criteria (p < 0.05) as defined in each dataset.

To ensure consistent and precise annotation, a custom-built mapping pipeline was implemented. At the protein level, gene symbols were first standardized using the HUGO Gene Nomenclature Committee (HGNC; https://www.genenames.org/) (accessed May 2023) database to ensure uniform and up-to-date gene nomenclature across all datasets. Subsequently, all MELK phosphosites were mapped to the canonical protein sequence using UniProt (https://www.uniprot.org/) (UniProt Consortium, 2023). Phosphosite information, including residue identity, position, and flanking sequence, was aligned to the corresponding UniProt protein sequence, with isoform-specific validation steps performed to resolve ambiguities and ensure accurate site assignment. This analytical framework is consistent with previously established large-scale phosphoproteomic integration studies.^20^, ^21^, ^22^

### 2.2 Identification of major MELK phosphosites

To pinpoint the primary regulatory phosphosites on MELK, we aggregated and analyzed all curated human phosphoproteomics datasets, quantifying the detection frequency of each phosphosite across studies. This analysis was performed separately for quantitative differential and qualitative profile datasets, with a focus on sites consistently reported in both serine/threonine- and tyrosine-enriched phosphoproteomes. Frequency distributions were compiled to identify high-recurrence phosphosites, which were designated as candidate regulatory nodes. For visualization and prioritization, lollipop plots were generated using the trackViewer R/Bioconductor package (https://doi.org/10.18129/B9.bioc.trackViewer). These plots integrated evidence from both dataset types, enabling intuitive ranking by detection frequency across diverse biological contexts.

Sites exhibiting the highest recurrence S356, S505, and S529, emerged as major phosphosites and were selected for downstream co-regulation, correlation, and network analyses due to their robust and reproducible detection across independent studies and experimental conditions.

### 2.3 Co-occurrence analysis of the MELK phosphosites

To explore the interrelationships among phosphosites in MELK, we performed a co-occurrence study, with a specific emphasis on the co-differential expression patterns of phosphosite pairs in this protein. We selected datasets showing differential regulation where several MELK phosphosites were identified under identical experimental settings. For every such pair, we computed the frequencies of UU (Up–Up), UD (Up–Down), DD (Down–Down), and DU (Down–Up) events individually, then evaluated positive co-regulation via the ratio ∑(nUU + nDD)/∑(nUD + nDU) and negative co-regulation via ∑(nUD + nDU)/∑(nUU + nDD).

### 2.4 Identification of high-confidence phosphosites in other proteins (PsOPs) co-regulated with MELK

To identify phosphosites on other proteins (PsOPs) that show positive or negative co-differential regulation with the major MELK phosphosites, differential regulation data for each phosphosite were examined individually across the available datasets; because the original studies were highly heterogeneous in experimental conditions, biological systems, and quantification methods, uniform reprocessing of raw data was not possible. Therefore, the identification of co-differentially regulated phosphosites and all subsequent analyses were performed using established methods from previous studies.Only phosphosites with a localization probability ≥ 75% and an A-score > 13 were considered for analysis to ensure high-confidence site detection and localisation.

Datasets were systematically organized according to the directional phosphorylation patterns of MELK phosphosites. Phosphosites in other proteins (PsOPs; denoted as “o”) that were downregulated or upregulated in response to MELK (denoted as “m”) upregulation were classified as DoUm and UoUm, respectively. Conversely, PsOPs that were upregulated or downregulated in response to MELK downregulation were categorized as UoDm and DoDm. PsOPs grouped as UoUm and DoDm were considered positively co-regulated (same-direction change), whereas those in DoUm and UoDm were classified as negatively co-regulated (opposite-direction change) relative to dynamics of MELK phosphosites.

To refine and prioritize candidates, Fisher’s exact test (FET) was applied for statistical evaluation. PsOPs were filtered using stringent criteria: a FET p-value < 0.05; a co-regulation ratio ≥ 15% (calculated as ∑(nUmUo + nDmDo)/∑(nUmDo + nDmUo) for positive co-regulation, and ∑(nUmDo + nDmUo)/∑(nUmUo + nDmDo) for negative co-regulation); evidence from at least two distinct experimental conditions (based on unique experimental identifiers); and support from a minimum of two independent publications (distinct PubMed IDs). To minimize false-positive associations resulting from dataset variability and ensure strong detection of consistent co-regulation patterns, an empirical cutoff of ≥ 15% was used for the co-regulation ratio. PsOPs meeting all these criteria were designated as high-confidence co-regulated phosphosites and were carried forward for downstream analyses.

### 2.5 Identification of potential upstream, phosphatases and binary interactors among the high confidence proteins

Following the identification of high-confidence phosphosites co-regulated with MELK, a comprehensive multi-level annotation workflow was employed to establish whether these sites are located on known or putative MELK substrates, direct interactors, or proteins phosphorylated by related kinases. Initially, experimentally validated kinase-substrate relationships and phosphorylation events were retrieved from curated resources, including PhosphoSitePlus (https://www.phosphosite.org/) (version 6.8.0, accessed 22 May 2023),^23^ Phospho.ELM (https://phospho.elm.eu.org/) (version 9.0, accessed 24 May 2023),^24^ and RegPhos (https://ngdc.cncb.ac.cn/databasecommons/database/id/665) (version 2.0, accessed 24 May 2023),^25^ thereby providing a solid foundation of empirical evidence for prioritizing biologically meaningful sites.

For phosphosites lacking prior experimental annotation, computational prediction tools were applied: NetworKIN (https://axis-shield-density-gradient-media.com/search.php) (predictions generated 04 Jan 2023)^26^ and Automatic Kinase-specific Interactions Detection (AKID) (predictions generated 24 May 2023)^27^ were used to infer candidate upstream kinases based on sequence motifs, contextual features, and co-regulation patterns (data accessed June 2023). These predictions were further supported by *in vitro* kinase–substrate relationships from In Vitro Kinase-to-Phosphosite Database (iKiP-DB) and refined using updated kinase-specific motif preferences reported by Johnson et al. (2023).^28^

To contextualize the phosphosites within broader signaling networks, protein–protein interaction data were integrated from multiple high-quality sources, including Human Protein Reference Database (HPRD; https://www.hprd.org/),^29^ Biological General Repository for Interaction Datasets (BioGRID; https://thebiogrid.org/),^30^ Berkeley Internet Name Domain (BIND; https://bio.tools/bind),^31^ ConsensusPathDB (https://cpdb.molgen.mpg.de/) (version 35, accessed 22 May 2023,^32^ and COmprehensive Resource of Mammalian protein complexes (CORUM) (https://mips.helmholtz-muenchen.de/corum/) (accessed 03 Mar 2023).^33^ Throughout the pipeline, all gene and protein identifiers were uniformly converted to official HGNC-approved symbols to guarantee accurate and consistent mapping of both regulatory and physical interactions.

### 2.6 Survival analysis

Survival analysis of few candidate genes MELK, MKI67 and TTK were performed using the Gene Expression Profiling Interactive Analysis 2 (GEPIA2) web server (https://gepia2.cancer-pku.cn/), a publicly accessible bioinformatics platform that integrates RNA sequencing expression data from The Cancer Genome Atlas (TCGA) and Genotype-Tissue Expression (GTEx) databases for interactive analysis of tumor and normal tissue gene expression. Gene expression and clinical survival data were retrieved from TCGA for three types of cancer: Breast Invasive Carcinoma (BRCA), Lung Adenocarcinoma (LUAD), and Liver Hepatocellular Carcinoma (LIHC). All data utilized within GEPIA2 are derived from level 3 TCGA RNA-seq data processed through a standardized pipeline using the UCSC Xena platform to ensure uniformity in normalization and batch correction across datasets.

Survival curves were constructed using the Kaplan-Meier (KM) method and visualized with 95% confidence interval bands represented as dotted lines flanking each survival curve. Between-group differences in survival were assessed using the log-rank test, with statistical significance set at p < 0.05. Hazard ratios (HR) and their associated p-values were estimated using Cox proportional hazards regression, with the High expression group designated as the reference comparator against the Low expression group. HR values greater than 1.0 indicated increased hazard (worse prognosis) in the High expression group. Both log-rank p-values and Cox regression p(HR) values are reported for each comparison. All statistical computations and graphical outputs were generated directly through the GEPIA2 platform without additional external statistical software.

### 2.7 Data visualisation

Regulatory and interaction networks were visualized using Cytoscape (v3.10.3)^34^ and RAWGraphs (https://www.rawgraphs.io/).

## 3 Results and Discussion

### 3.1 Global phosphoproteome atlas and major phosphosites of MELK

After reviewing 3,825 publicly available mass-spectrometry-based global phosphoproteomic datasets from human cell studies, 739 qualitative profiling datasets and 148 quantitative differential datasets associated with MELK were identified. From the qualitative profiling datasets, a total of 68 unique MELK phosphosites were systematically compiled, while the quantitative differential datasets indicated 21 MELK phosphosites that displayed differential regulation. The complete dataset is provided in the Supplementary Table (S1, S2). The phosphosites S356, S505, and S529 were the most frequently detected among these datasets, emerging as the major regulatory sites of MELK.

**Fig. 1:**
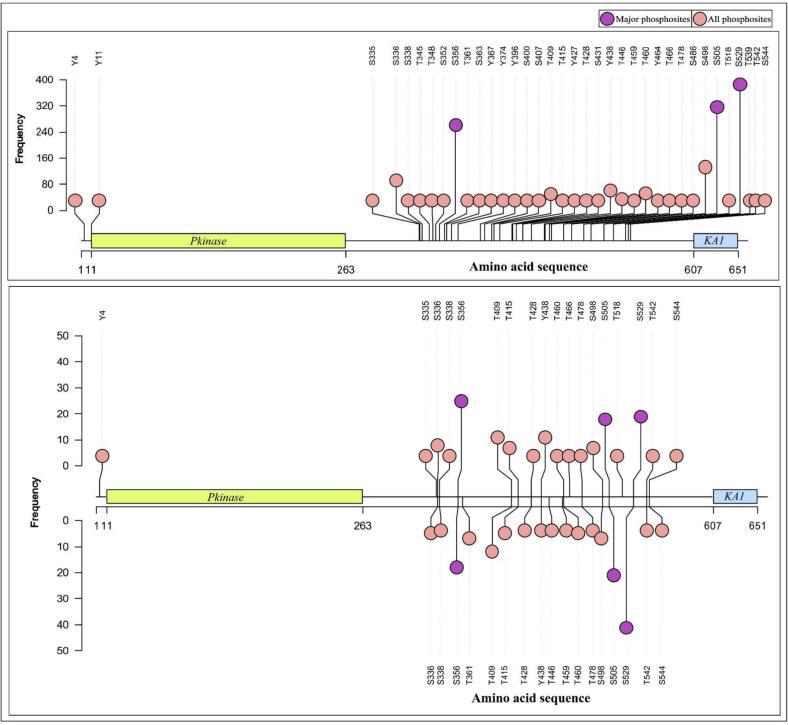
**Distribution of MELK phosphosites.** The lollipop plot illustrates the annotated domains of MELK (Pkinase, KA1) with identified phosphosites. Major sites S356, S505, and S529 are marked in purple, indicating their frequent detection in global phosphoproteomic datasets. (For interpretation of the references to colour in this figure legend, the reader is referred to the web version of this article.)

To illustrate their distribution, we created a lollipop plot of the MELK protein sequence (Fig. 1). This plot displays the frequency and spatial distribution of all known MELK phosphosites (pink circles) along the protein sequence. The three most frequently observed sites. are highlighted in purple circles. Annotated domains, such as the kinase domain (residues 11–263) and the KA1 domain (residues 607–651), offer structural context. These findings indicate that S356, S505, and S529 are functionally significant phosphosites. This spatial clustering of major phosphosites outside the canonical kinase domain suggests their importance in non-catalytic regulatory roles, perhaps influencing interactions, localization, or substrate specificity.

### 3.2 Co-occurrence pattern of MELK phosphosites

**Fig. 2:**
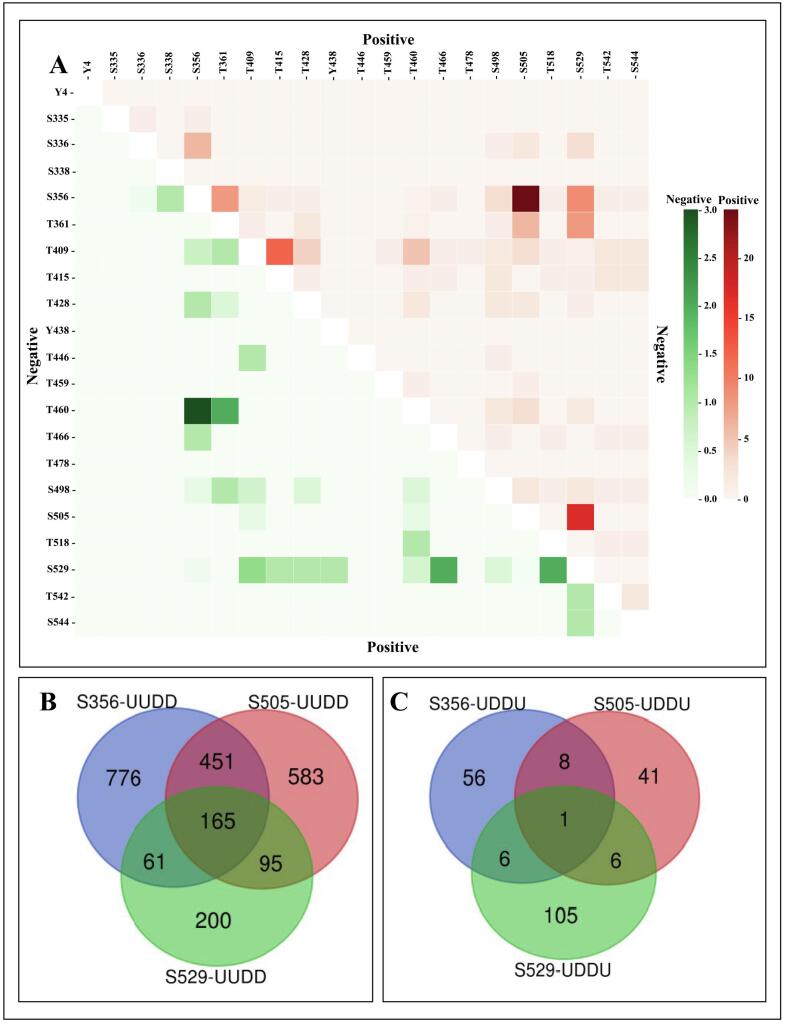
**Co-occurrence of MELK phosphosites.** (A) The heatmap displays positive (UUDD, red) and negative (UDDU, green) co-occurrence patterns among MELK sites. (B) Venn diagrams depict phosphosites positively and negatively co-regulated across S356, S505, and S529. Collectively, these analyses demonstrate that S356, S505, and S529 constitute a closely connected phosphorylation triad. (For interpretation of the references to colour in this figure legend, the reader is referred to the web version of this article.)

Integrative co-occurrence analysis, presented in Fig. 2**A** as a heatmap of pairwise phosphosite co-occurrence, revealed striking patterns of both positive and negative correlations across 21 detected MELK phosphosites. The most prominent feature was a strong positive co-occurrence between S356 and S505 (dark red), indicating near-simultaneous phosphorylation of these sites. Moderate positive co-occurrence was also observed between S529 and S505 (orange-red), and between T415 and T409, suggesting a functionally linked phosphorylation cluster in the central region of the protein. In contrast, T460 exhibited a strong negative correlation with S356 (dark green) and a moderate negative correlation with T361, indicating mutually exclusive phosphorylation events, while S529 exhibited moderate negative correlation with T466 and T518 Lower MELK sites (e.g., S544, T542, T518) showed minimal co-occurrence with others, appearing mostly white or light pink, suggesting independent or weakly coupled regulation.Venn diagram analysis further demonstrated substantial overlap of co-regulated phosphosites (PsOPs) in the positive co-regulation condition (UUDD), with 451 PsOPs shared between S356 and S505 (Fig. 2**B**). These patterns support the existence of cooperative (S356-S505) and partially antagonistic modules in MELK regulation.

To further validate these relationships under differential regulatory conditions, we analyzed phosphopeptide abundance in two contrasting states: UUDD (upregulated in upregulated conditions, downregulated in downregulated) and UDDU (upregulated in downregulated conditions, downregulated in upregulated) using Venn diagram analysis (Fig. 2**B and** Fig. 2**C**) In the UUDD condition (Fig. 2**B**), S356-UUDD and S505-UUDD shared the largest overlap of 451 PsOPs, reinforcing their co-regulation. A smaller but significant overlap existed between S356-UUDD and S529-UUDD (61 PsOPs), as well as between S505-UUDD and S529-UUDD (95 PsOPs). In total, 165 PsOPs were common across all three sites, defining a core co-regulated module. In contrast, the UDDU condition (Fig. 2**C**) showed dramatically reduced overlap, with only 1 PsOP common to all three sites and pairwise overlaps ranging from 6 to 8 PsOPs.

In summary, MELK phosphorylation is not uniform but likely organized into cooperative (S356-S505-T409) and antagonistic (T460) modules. These findings suggest that MELK activity is finely tuned by multi-site phosphorylation logic, enabling context-dependent responses in cell proliferation, survival, or stress adaptation. Future studies should employ site-specific mutagenesis and kinase profiling to delineate the upstream regulators and functional consequences of these phosphosite networks.

### 3.3 Co-regulated phosphoproteins (PsOPs) with MELK major sites

Identification of high confidence pPsOPs that showed co-regulation with the major MELK phosphosites using the criteria mentioned in section 2.4 uncovered extensive co-regulation. MELK S356 co-regulated with 1,454 positively and 71 negatively co-regulated PsOPs, S505 with 1,294 positive and 56 negative co-regulated PsOPs, and S529 with 521 positive and 118 negative co-regulated PsOPs (Supplementary Table: S3-S8).

**Fig. 3:**
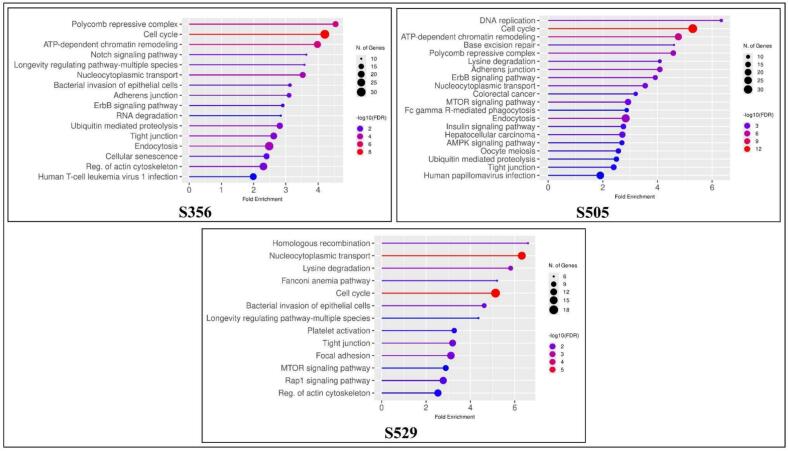
**Pathway enrichment analysis of co-regulated n proteins associated with the three major MELK phosphosites S356, S505, and S529.** Lollipop plots illustrate KEGG pathways that are significantly enriched, with fold enrichment shown on the x-axis, the size of the bubbles representing the number of genes, and the color of the bubbles indicating statistical significance (–log10 FDR). PsOPs associated with S356 are enriched in chromatin remodeling, cell cycle, and junction organization; PsOPs connected to S505 demonstrate notable enrichment in DNA replication, cell cycle, AMPK/MTOR signaling, and cancer-related pathways; while PsOPs related to S529 are enriched in homologous recombination, the Fanconi anemia pathway, focal adhesion, and signaling associated with the cytoskeleton.

In order to better understand the functional landscape linked to the primary phosphosites of MELK, KEGG pathway enrichment analysis was conducted using ShinyGo^35^ on their positively co-regulated PsOPs networks. The module associated with S356 demonstrated significant enrichment for pathways related to chromatin remodeling, control of the cell cycle, Notch and ErbB signaling, RNA degradation, transport between the nucleus and cytoplasm, as well as various junctional and cytoskeletal processes, suggesting that this site plays a role in coordinating chromatin architecture and intercellular organization. On the other hand, DNA replication, cell-cycle progression, base excision repair, MTOR/AMPK signaling, lysine degradation, Fc-gamma receptor-mediated phagocytosis, and several cancer-associated pathways dominated the S505 PsOPs network's wider and broad enrichment signature, indicating S505 as the oncogenic regulatory node. The PsOPs associated with S529 showed a distinct enrichment pattern that was more selective, focusing on pathways such as homologous recombination, Fanconi anemia, nucleocytoplasmic transport, focal adhesion, regulation of the actin cytoskeleton, Rap1, and MTOR signaling. This indicates their involvement in maintaining the genome and remodeling the cytoskeleton. When taken as a whole, the phosphosite-resolved enrichment patterns show that S356, S505, and S529 create three functionally different MELK signaling modules: proliferative signaling, DNA repair–cytoskeletal programming, and chromatin control. The results are summarized in Fig. 3, with S356 (top left), S505 (top right), and S529 (bottom) panels.

### 3.4 Predicted upstream kinases among the co-regulated MELK phosphosites

**Fig. 4:**
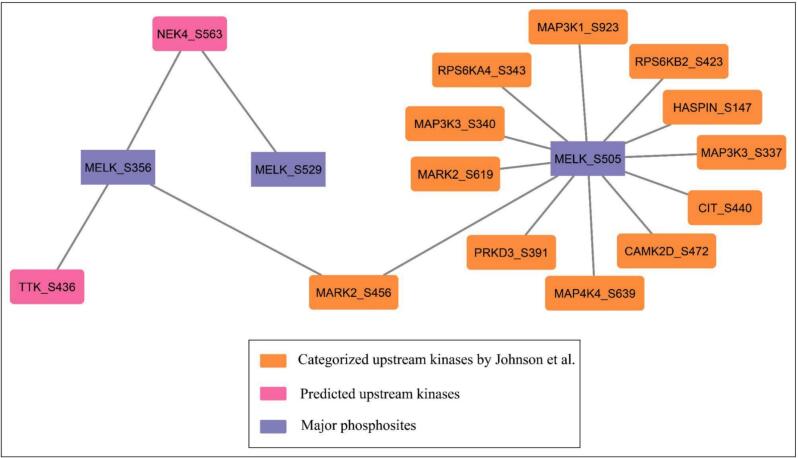
**Upstream kinases of MELK phosphosites.** Network representation of categorized and predicted upstream kinases regulating major MELK phosphosites (S356, S505, S529). Major MELK phosphosites are shown in purple, categorized upstream kinases (Johnson et al.) in orange, and predicted upstream kinases in pink. (For interpretation of the references to colour in this figure legend, the reader is referred to the web version of this article.)

A total of 14 upstream kinases were predicted for the major identified phosphosites of MELK, as illustrated in Fig. 4, where major phosphosites (S356, S505, S529) are shown as central nodes connected to their respective upstream kinases. Among them, serine/threonine-protein kinase MARK2 (MARK2) was identified as a potential positive upstream kinase of both MELK_S356 at site S456 and MELK_S505 at site S619, suggesting site-specific regulation. At S505, additional positively co-regulated upstream kinases included mitogen-activated protein kinase kinase kinase 1 (MAP3K1_S923), serine/threonine-protein kinase haspin (HASPIN_S147), serine/threonine-protein kinase D3 (PRKD3_S391), mitogen-activated protein kinase kinase kinase 3 (MAP3K3_S337/S340), citron Rho-interacting kinase (CIT_S440), mitogen-activated protein kinase kinase kinase kinase 4 (MAP4K4_S639) and ribosomal protein S6 kinase beta-2 (RPS6KB2_S423). Calcium/calmodulin-dependent protein kinase type II subunit delta (CAMK2D_S472) and ribosomal protein S6 kinase alpha-4 (RPS6KA4_S343) were negatively co-regulated (Supplementary Table- S9). These results imply that MELK phosphorylation is modulated by a wide array of upstream regulators, spanning MAPK, AGC, CaMK, and cytoskeleton-associated kinase families, reflecting the complexity of its kinase signaling network.

In addition to experimentally identified upstream kinases, predictive analyses revealed that serine/threonine-protein kinase Nek4 (NEK4) and dual specificity protein kinase TTK (TTK, also known as Mps1) were potential kinases. NEK4 (S563) was predicted to positively co-regulate with MELK at S356 and S529, while TTK (S436) was predicted to positively co-regulate MELK at S356 **(**Fig. 4**,** Supplementary Table-S10). Both kinases are tightly linked to mitotic control and spindle assembly checkpoint signaling, with NEK4 functioning in microtubule organization and mitotic progression^36^ and TTK serving as a critical mitotic checkpoint kinase ensuring accurate chromosome segregation.^37^ This association suggests that MELK may be regulated by pathways important for spindle assembly and chromosomal stability, supporting MELK’s broader roles in cell cycle progression and genome integrity, in line with its well-characterized function in mitosis.^38^

The dominance of categorized kinases (all from Johnson et al.) converging on S505-including members of the MAPK (MAP3K1, MAP3K3), RSK (RPS6KA4, RPS6KB2), CaMK (CAMK2D), and Hippo-related (MAP4K4) families-suggests that S505 might serve as a convergence point for mitogenic, stress, and cell cycle signals. This is consistent with its strong co-phosphorylation with S356 (Fig. 2**A)** and extensive peptide overlap in UUDD conditions (Fig. 2**B**), implying that S505 phosphorylation may prime or stabilize S356 modification, possibly via conformational exposure or scaffold recruitment.

The exclusive reliance of S356 on predicted kinases (NEK4, TTK) is striking. Both NEK4 and TTK (MPS1) are mitotic kinases implicated in centrosome and spindle checkpoint regulation, aligning with MELK’s known roles in mitosis. The lack of shared kinases between S356, S505, and S529-despite co-occurrence patterns-implies non-overlapping kinase-substrate recognition or sequential phosphorylation logic. For instance, S505 may be a priming event that enables NEK4/TTK access to S356.

In summary, MELK is controlled by a tiered kinase network: S505 as a multivalent activation hub along with S356 under mitotic-specific predicted control. These findings reconcile co-occurrence patterns with kinase specificity and suggest that MELK integrates diverse signals through spatially and temporally segregated phosphosites. Future work should validate NEK4/TTK − S356 axes using kinase inhibition and phosphosite-specific MELK mutants in mitotic models.

### 3.5 Co-regulated kinases associated with MELK major phosphosites

Co-regulation analysis of MELK kinases revealed 80 positively and 3 negatively co-regulated PsOPs with S356, 72 positively and 4 negatively co-regulated PsOPs with S505, and 34 positively and 4 negatively co-regulated PsOPs with S529 (Supplementary Table- S11). Among these, citron Rho-interacting kinase (CIT) at S440, eukaryotic elongation factor 2 kinase (EEF2K) at S18, non-receptor tyrosine-protein kinase (TYK2) at Y292, phosphatidylinositol 4-kinase alpha (PI4KA) at S265, and cAMP-dependent protein kinase type I-beta regulatory subunit (PRKAR1B) at S83 consistently exhibited co-regulation across all three MELK phosphosites. These kinases participate in diverse signaling pathways such as cytoskeletal organization (CIT),^39^ translational regulation (EEF2K),^40^ cytokine-driven JAK/STAT signaling (TYK2),^41^ phosphoinositide metabolism (PI4KA),^42^ and cAMP/PKA signaling (PRKAR1B).^43^ This conserved co-regulatory pattern suggests the presence of core signaling modules engaged by MELK during various activation states, underscoring its multifaceted regulatory role in key cellular pathways.

**Fig. 5:**
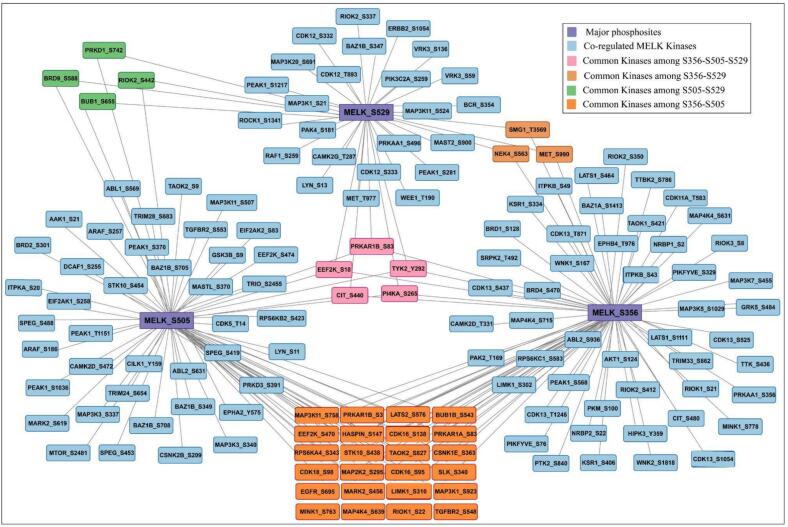
**Co-regulated kinases associated with MELK major phosphosites.** Purple nodes represent MELK sites, while connected nodes denote co-regulated kinases. Color coding marks overlapping kinases: pink (common to all three sites), orange (S356–S529), green (S505–S529), and brownish orange (S356–S505), highlighting both site-specific and shared regulatory partners. (For interpretation of the references to colour in this figure legend, the reader is referred to the web version of this article.)

Similarly, 29 kinases were found to be common between S356 and S505 including: Mitogen-activated protein kinase kinase kinase 1 (MAP3K1_S758), Serine/threonine-protein kinase MARK2 (MARK2_S456), LIM domain kinase 1 (LIMK1_S310), MAP3K1_S923, Serine/threonine-protein kinase RIO1 (RIOK1_S22), TGF-beta receptor type-2 (TGFBR2_S548), Misshapen-like kinase 1 (MINK1_S763), Mitogen-activated protein kinase kinase kinase kinase 4 (MAP4K4_S639), Epidermal growth factor receptor (EGFR_S965), cAMP-dependent protein kinase type I-alpha regulatory subunit (PRKAR1A_S83), Serine/threonine-protein kinase 10 (STK10_S458). These proteins are respectively involved in key cellular processes: MAP3K1 in signaling pathways regulating cell survival, apoptosis, and migration^44^; MARK2 in establishing cell polarity and regulating microtubule dynamics^45^; LIMK1 in actin cytoskeleton remodeling critical for cell migration and morphology^46^; RIOK1 in ribosome biogenesis and cellular adaptation^47^; TGFBR2 in TGF-β-mediated control of cell growth and differentiation^48^; MINK1 in neuronal signaling and synaptic function regulation^49^; MAP4K4 in stress-activated MAPK pathways and cytoskeletal remodeling^50^; EGFR in receptor tyrosine kinase signaling driving proliferation and survival^51^; PRKAR1A functions as a dual tumor regulator, influencing cell proliferation in cancer cells and maintaining stemness in cancer stem cells through varying ERK-pathway signaling^52^ and STK10 in immune cell signaling and cytoskeletal dynamics modulation.^53^ These common proteins define the S356–S505 co-phosphorylation axis observed in co-occurrence analysis (Fig. 5). For S356 and S529, a total of 9 kinases were identified, which include: Serine/threonine-protein kinase SMG1 (SMG1_T3669), Serine/threonine-protein kinase Nek4 (NEK4_S563), Hepatocyte growth factor receptor (MET_S990), were shared, linking stress responsive signaling to this site. SMG1 functions as a PIKK kinase that responds to stress by coordinating DNA damage signaling through the activation of p53 in reaction to double-strand breaks.^54^ NEK4 functions as a kinase related to NIMA that responds to DNA damage, controlling replicative senescence and non-homologous end joining by facilitating the recruitment of DNA-PK and the activation of p53.^55^ MET functions as a receptor tyrosine kinase regulated by integrated stress responses, promoting oncogenic overexpression in response to cellular stress.^56^

Also, for S505 and S529, there are 10 kinases including: Serine/threonine-protein kinase D1 (PRKD1_S742), Mitotic checkpoint serine/threonine-protein kinase BUB1 (BUB1_S655), Serine/threonine-protein kinase RIO2 (RIOK2_S442), in which are involved in mitotic regulation. BUB1 functions as a fundamental kinase in the mitotic checkpoint, influencing the assembly of kinetochores, the sensing of spindle attachment, and the accuracy of chromosome segregation.^57^ RIOK2 acts as a kinase-like factor regulated by PLK1, influencing the timing of the transition from metaphase to anaphase and the exit from mitosis.^58^ PRKD1 functions as a Ser/Thr kinase associated with the centrosome and spindle, playing a role in regulating the transition from metaphase to anaphase and ensuring the accuracy of chromosome segregation.^59^

In summary, MELK phosphosites are embedded in a highly interconnected co-regulatory kinase web with S356 as a mitotic effector, and S529 as a polarity integrator. The S356-S505 axis could potentially be the dominant co-regulated module, underpinned by MAPK/Hippo convergence, while S529 maintains partial linkage via stress/mitotic kinases.

### 3.6 Phosphatases positively co-regulated with major phosphosites

**Fig. 6:**
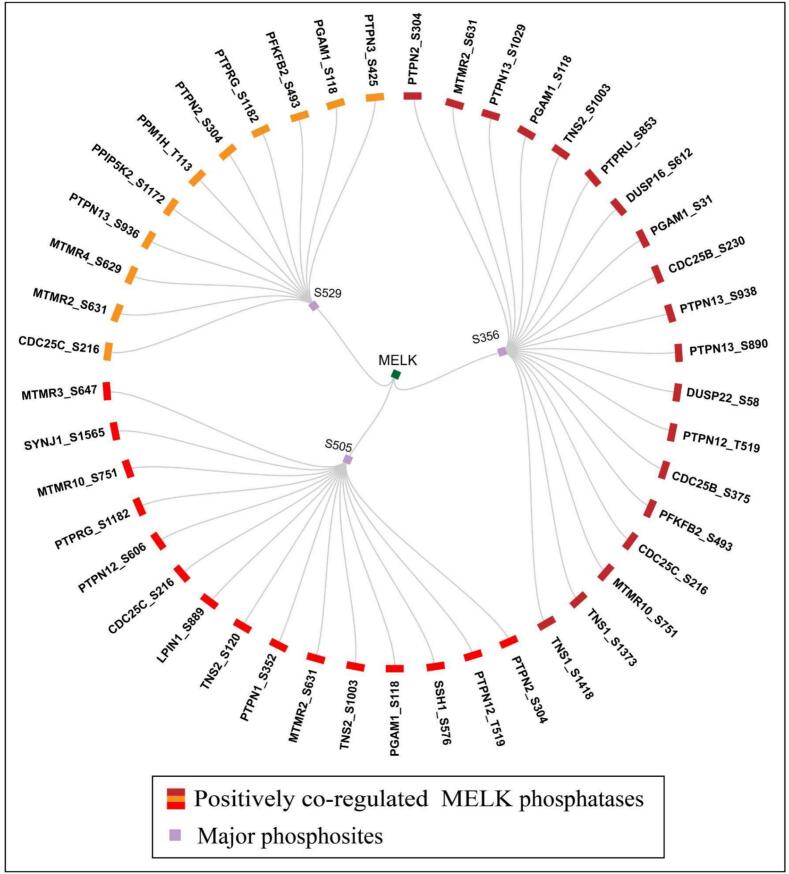
**MELK phosphatases positively co-regulated with major MELK phosphosites.** Circular network representation showing phosphatases (outer ring) positively co-regulated with MELK at major phosphosites (S356, S505, S529). The coloured bars indicate positively co-regulated MELK phosphatases, while the purple node marks the major MELK phosphosites. (For interpretation of the references to colour in this figure legend, the reader is referred to the web version of this article.)

For MELK, we identified 19 positively co-regulated PsOPs at S356, 15 PsOPs at S505, and 11 PsOPs at S529 (Fig. 6**,** Supplementary Table-S12). The phosphatase co-regulation network unveils a finely tuned dephosphorylation hierarchy that mirrors and counterbalances the kinase inputs, with S505 under extensive reversible control, S356 under mitotic-specific feedback, and S529 under lipid- and endocytosis-linked regulation. Four phosphatases: protein tyrosine phosphatase non-receptor type 2 (PTPN2_S304), myotubularin-related protein 2 (MTMR2_S631), phosphoglycerate mutase 1 (PGAM1_S118), and cell division cycle 25C (CDC25C_S216), were consistently associated with all three MELK sites (S356, S505, S529), indicating their role as core regulators of MELK dephosphorylation.

At S356, additional associations included: protein tyrosine phosphatase non-receptor type 13 (PTPN13_S1029/S938/S890), PGAM1_S31, tensin-2 (TNS2_S1003), protein tyrosine phosphatase receptor type U (PTPRU_S853), dual specificity phosphatase 16 (DUSP16_S612), cell division cycle 25B (CDC25B_S230/S375), dual specificity phosphatase 22 (DUSP22_S58), protein tyrosine phosphatase non-receptor type 12 (PTPN12_T519), 6-phosphofructo-2-kinase/fructose-2,6-bisphosphatase 2 (PFKFB2_S493), myotubularin-related protein 10 (MTMR10_S751), and tensin-1 (TNS1_S1373/S1418). At S505, MELK was further linked with PTPN12_T519/S606, slingshot protein phosphatase 1 (SSH1_S576), TNS2_S1003/S120, protein tyrosine phosphatase non-receptor type 1 (PTPN1_S352), lipin-1 (LPIN1_S889), protein tyrosine phosphatase receptor type G (PTPRG_S1182), MTMR10_S751, synaptojanin-1 (SYNJ1_S1565), and myotubularin-related protein 3 (MTMR3_S647). At S529, additional associations included myotubularin-related protein 4 (MTMR4_S629), PTPN13_S936, diphosphoinositol pentakisphosphate kinase 2 (PPIP5K2_S1172), protein phosphatase 1H (PPM1H_T113), PTPRG_S1182, PFKFB2_S493, and protein tyrosine phosphatase non-receptor type 3 (PTPN3_S425).

Functionally, these phosphatases include classical tyrosine phosphatases (PTPN family, PTPRG, PTPRU),^60^ dual-specificity MAPK phosphatases (DUSP16, DUSP22) (Seternes et al. 2019), cell-cycle phosphatases (CDC25B/C),^61^ lipid-directed phosphatases (MTMR family, SYNJ1),^62^ and serine/threonine phosphatases or regulators (PPM1H, SSH1).^63^ The involvement of metabolic regulators such as PGAM1 and PFKFB2, along with phosphoinositide-directed enzymes like MTMRs, SYNJ1, and PPIP5K2, further suggests an interaction between MELK phosphorylation and metabolic or membrane-associated signaling. Overall, these findings imply that MELK is governed by a core group of phosphatases, supplemented by site-specific enzymes that adjust its phosphorylation state within cell-cycle, MAPK, metabolic, and lipid signaling networks.

The phosphatase co-regulation network establishes a multi-layered dephosphorylation logic that precisely counterbalances the kinase hierarchy **(**Fig. 4, Fig. 5) and reinforces functional segregation of MELK phosphosites.

### 3.7 Binary interactor phosphosite co-regulation network identifies core and site-specific MELK interactors

**Fig. 7:**
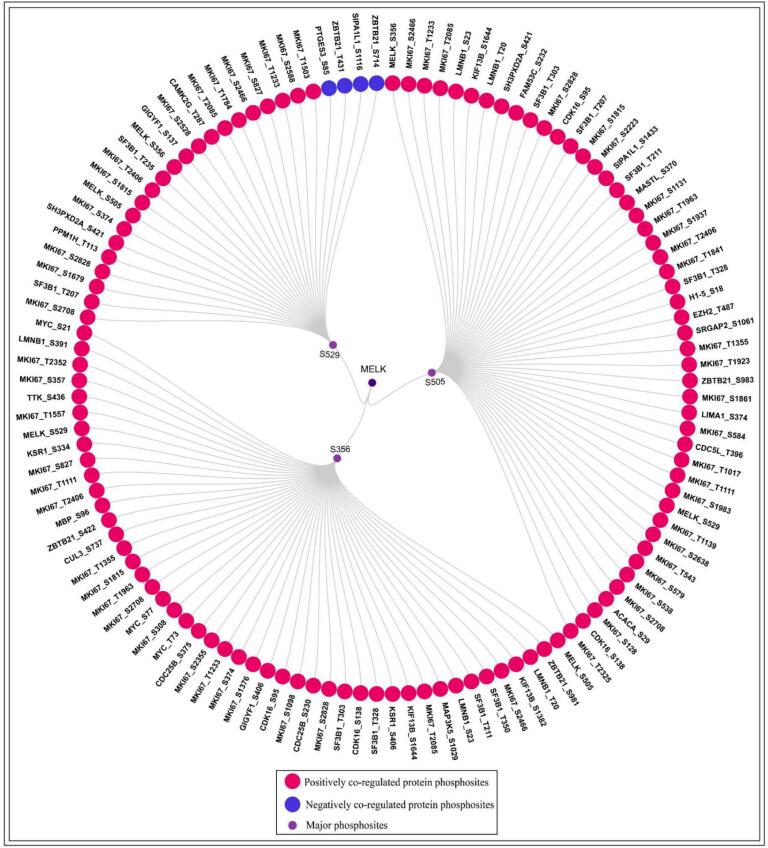
**MELK interactors derived from binary phosphosite co-regulation and curated signaling networks.** Circular diagram illustrating phosphosites associated with MELK. Major MELK sites S356/S505/S529 are highlighted in purple, with sites showing positive co-regulation in pink and those with negative co-regulation in blue. (For interpretation of the references to colour in this figure legend, the reader is referred to the web version of this article.)

To define the functional interactome of MELK at the level of binary phosphosite co-regulation, we mapped 120 PsOPs across partner proteins that show statistically significant co-variation with major sites of MELK **(**Fig. 7**)**. These comprised 46 positively co-regulated PsOPs with S356, 48 PsOPs with S505, and 22 PsOPs positively, along with 4 negatively co-regulated PsOPs with S529, revealing extensive phosphodynamic coupling between MELK and its regulatory network (Supplementary Table- S13).

A core set of seven MKI67 (Proliferation marker protein Ki-67) phosphosites-T2085, S2466, T2406, S2828, T1233, S1815, and S2708-emerged as pan-site co-regulators, marking MKI67, a nuclear protein essential for ribosomal RNA synthesis and a hallmark of proliferating cells, as the central, proliferation-linked partner of MELK across all activation states. Pairwise overlaps further revealed functional specificity: S356 ∩ S505 (n = 19) included MKI67_T1355/T1111, Kinesin-like protein KIF13B (KIF13B_S1644) (a kinesin mediating mitotic spindle orientation), Splicing factor 3B subunit 1 (SF3B1_T303/T328/T211) (a core spliceosome component regulating alternative splicing),^64^ Cyclin-dependent kinase 16 (CDK16_S138/S95) (a PCTAIRE kinase involved in vesicle transport and neurite outgrowth),^65^ and Lamin-B1 (LMNB1_T20/S23) (lamin B1, a nuclear lamina scaffold critical for mitotic spindle matrix assembly)^66^; S356 ∩ S529 (n = 10) featured MKI67_S374/S827, reinforcing MKI67′s context-dependent linkage; and S505 ∩ S529 (n = 10) comprised SF3B1_T207 and SH3 and PX domain-containing protein 2A (SH3PXD2A_S421) (Tks5, an adaptor in podosome formation and invadopodia during cancer invasion).

Site-exclusive interactors underscored modular regulation: S356 was uniquely coupled to Dual specificity protein kinase TTK (TTK_S436) (MPS1, spindle assembly checkpoint kinase),^37^ Mitogen-activated protein kinase kinase kinase 5 (MAP3K5_S1029) (ASK1, stress-activated kinase),^67^ Kinase suppressor of Ras 1 (KSR1_S406/S334) (scaffold for RAF/MEK/ERK),^68^ M−phase inducer phosphatase 2 (CDC25B_S230/S375) (G2/M phosphatase),^69^ Cullin-3 (CUL3_S737) (E3 ubiquitin ligase subunit),^70^ Myc proto-oncogene protein (MYC_T73/S77/S21) (proto-oncogene driving proliferation),^71^ and Myelin basic protein (MBP_S96) (myelin basic protein, structural regulator),^72^ collectively promoting mitotic entry and chromatin licensing; S505 (n = 48) linked to Histone-lysine N-methyltransferase EZH2 (EZH2_T487) (PRC2 catalytic subunit enforcing H3K27me3 silencing),^73^ Cell division cycle 5-like protein (CDC5L_T396) (spliceosome activator),^74^ Serine/threonine-protein kinase greatwall (MASTL_S370) (Greatwall kinase ensuring mitotic phosphatase inhibition),^75^ LIM domain and actin-binding protein 1 (LIMA1_S374) (EPLIN, actin stabilizer at spindle poles),^76^ and Signal-induced proliferation-associated 1-like protein 1 (SIPA1L1_S1433) (RapGAP modulating cytoskeletal dynamics),^77^ supporting chromatin compaction and spindle fidelity; and S529 associated with Calcium/calmodulin-dependent protein kinase type II subunit gamma (CAMK2G_T287) (calcium/calmodulin-dependent kinase in stress signaling),^78^ GRB10-interacting GYF protein 1 (GIGYF1_S137) (translational repressor in stress granules),^79^ Zinc finger and BTB domain-containing protein 21 (ZBTB21_T431/S714) (zinc-finger transcription repressor),^80^ and Prostaglandin E synthase 3 (PTGES3_S85) (p23 co-chaperone in prostaglandin synthesis) implicating stress adaptation, polarity, and metabolic-inflammatory integration.

Notably, S529 alone showed negative co-regulation with PTGES3_S85, ZBTB21_T431, SIPA1L1_S1116, ZBTB21_S714 indicating checkpoint-mediated suppression during stress or quiescence. Collectively, these binary interactions position MELK as a phosphorylation orchestrator bridging mitotic progression (MKI67, CDC25B, TTK), RNA/chromatin biology (SF3B1, EZH2, MYC), cytoskeletal dynamics (LMNB1, LIMA1), and adaptive signaling (CAMK2G, PTGES3), with MKI67 as the universal proliferative anchor and site-specific modules enabling context-dependent control in proliferation, mitosis, and stress response.

In summary, the integrated phosphoproteomic analyses establish MELK as a highly modular signaling hub governed by three functionally distinct phosphosites: S356 and S505 as a tightly co-regulated mitotic engine driving spindle assembly, chromatin organization, and proliferative gene expression through shared interactors such as MKI67, CDC25B, and EZH2; S529 as a stress- and polarity-responsive sensor integrating calcium, metabolic, and inflammatory cues via unique partners like CAMK2G and PTGES3; and MKI67 as the universal co-regulator anchoring MELK activity to cellular proliferation. Supported by convergent kinase inputs, phosphatase counter-regulation, and directional signaling loops, this site-resolved regulatory framework elucidates MELK’s oncogenic versatility and provides a molecular blueprint for precision therapeutic targeting in hyperproliferative and stress-adapted malignancies.

### 3.8 Downstream substrates among the co-regulated proteins

**Fig. 8:**
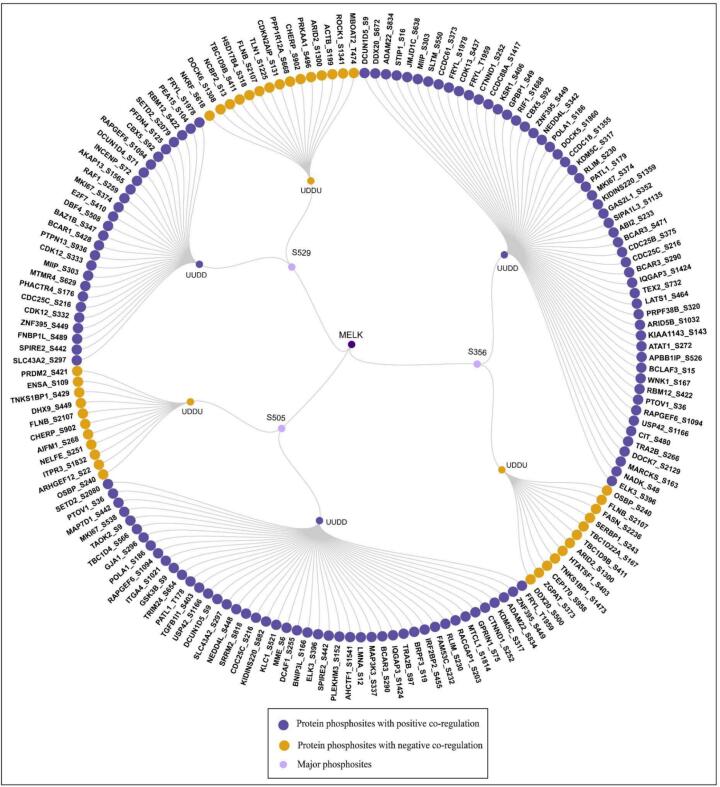
**Circular map depicting MELK co-regulated substrates associated with major phosphosites.** Central purple nodes denote MELK major phosphosites, while outer nodes represent substrate phosphosites. Substrates with positive co-regulation are highlighted in dark purple, and those with negative co-regulation in yellow, illustrating the balance of activating and inhibitory regulatory interactions mediated by MELK. (For interpretation of the references to colour in this figure legend, the reader is referred to the web version of this article.)

We identified 166 substrates (Fig. 8), comprising 54 positively and 12 negatively co-regulated PsOPs with S356, 46 positively and 11 negatively co-regulated PsOPs with S505, and 27 positively and 15 negatively co-regulated PsOPs with S529 (Supplementary Table- S14). A core substrate triad- M−phase inducer phosphatase 3 (CDC25C_S216), Zinc finger protein 395 (ZNF395_S449), and Rap guanine nucleotide exchange factor 6 (RAPGEF6_S1094), was consistently co-regulated across all three MELK sites.

Pairwise overlaps revealed 15 substrates shared between S356 and S505 including E3 ubiquitin-protein ligase RLIM (RLIM_S230), Ubiquitin carboxyl-terminal hydrolase 42 (USP42_S1166), Prostate tumor-overexpressed gene 1 protein (PTOV1_S36), Ras GTPase-activating-like protein IQGAP3 (IQGAP3_S1424), Catenin delta-1 (CTNND1_S252), ETS domain-containing protein Elk-3 (ELK3_S396), Lysine-specific demethylase 5C (KDM5C_S317), and DNA polymerase alpha catalytic subunit (POLA1_S186), 8 between S356 and S529 including Chromobox protein homolog 5 (CBX5_S92), Proliferation marker protein Ki-67 (MKI67_S374), and RNA-binding protein 12 (RBM12_S422), and 5 between S505 and S529, Protein spire homolog 2 (SPIRE2_S442) and Large neutral amino acids transporter small subunit 4 (SLC43A2_S297). Site-exclusive substrates were enriched in cell cycle regulators (e.g., CDC25C, GAS2L1, CBX5 at S356; CDC25C, GSK3B at S505),^81^, ^82^ chromatin modifiers (e.g., CBX5 at S356–S529),^83^ splicing factors (e.g., PRPF38B at S356, SRRM2 at S505)^84^, signaling kinases (e.g., GSK3B S505, RAF1 S529, ROCK1 at S529),^85^, ^86^ cytoskeletal components (e.g., FLNB at S356, S505 and S529),^87^ and metabolic enzymes (e.g., FASN, NADK at S356).^88^ Positive co-regulation, predominantly at S529, involved inhibitory sites on CDK12(S529), CDK13(S356), and MKI67 at S356, S505, S529. The high-throughput substrate NKRF_S618 (Negative co-regulation-S529) was also confirmed.

**Fig. 9:**
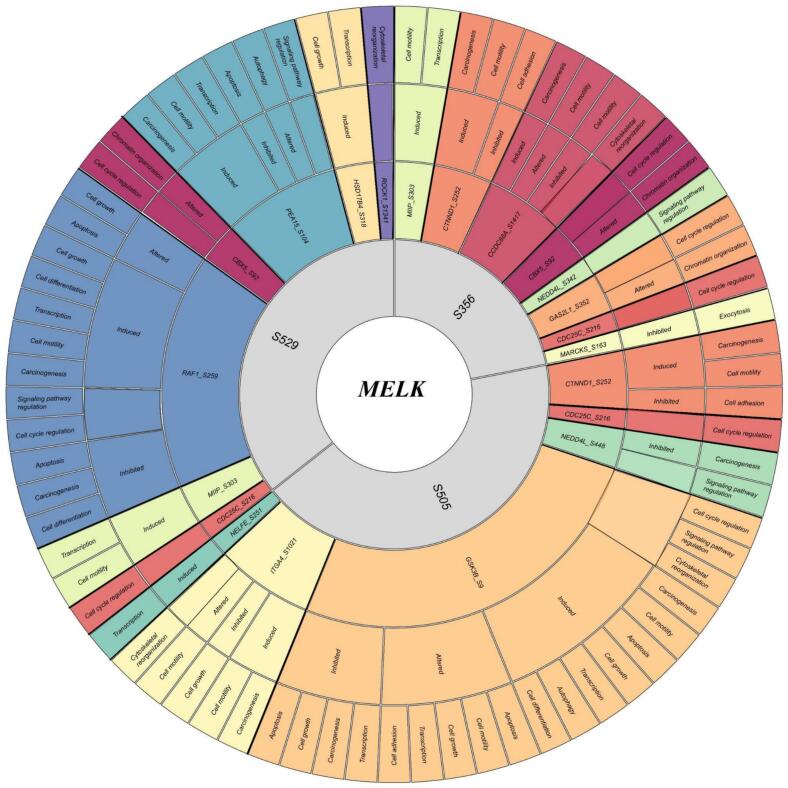
**Sunburst representation of MELK substrates and their related biological processes.** The circular diagram depicts the biological functions linked to the identified MELK substrates. This chart is categorized by major phosphosite (S356, S505, S529). Each section provides information about a specific co-regulated substrate, the type of regulatory effect (induced, inhibited, or altered), and the associated downstream biological processes, including carcinogenesis, cell cycle regulation, and apoptosis.

Functional enrichment analysis of MELK co-regulated substrates and interactors, visualized in a radial pathway map (Fig. 9), revealed site-specific orchestration of cellular processes centered on MELK’s major phosphosites (S356, S505, S529). S356 was linked to induced cell cycle regulation, carcinogenesis, cell motility, apoptosis inhibition, transcription, and chromatin organization, with key substrates including M−phase inducer phosphatase 3 (CDC25C_S216) (induced cell cycle),^88^ E3 ubiquitin-protein ligase NEDD4-like (NEDD4L_S448) (signaling pathway regulation),^89^ GAS2-like protein 1 (GAS2L1_S352), Chromobox protein homolog 5 (CBX5_S92) (altered chromatin),^90^ Catenin delta-1 (CTNND1_S252) (adhesion),^91^ Myristoylated alanine-rich C-kinase substrate (MARCKS_S163) (exocytosis). S505 featuring substrates like Negative elongation factor E (NELFE_S251), CDC25C_S216, Catenin delta-1 (CTNND1_S252), NEDD4L_S448, Glycogen synthase kinase-3 beta (GSK3B_S9) and Integrin alpha-4 (ITGA4_S1021) were involved in induced transcription, cell growth, cytoskeletal reorganization, cell motility, and carcinogenesis etc. S529 was associated with CDC25C_S216, Migration and invasion-inhibitory protein (MIIP_S303), RAF proto-oncogene serine/threonine-protein kinase (RAF1_S259), CBX5_S92, Astrocytic phosphoprotein PEA-15 (PEA15_S104), Peroxisomal multifunctional enzyme type 2 (HSD17B4_S318), Rho-associated protein kinase 1 (ROCK1_S1341) and were involved in biological processes like inhibited apoptosis, altered cell cycle regulation, induced transcription, and altered signaling pathways. Cross-site convergence was observed at CDC25C_S216 (shared between S356 and S505, inducing cell cycle progression) and CBX5_S92 (altered in both S356 and S529, affecting chromatin organization). Pathway polarity showed S356 and S505 predominantly inducing pro-oncogenic programs (cell cycle, motility, survival), while S529 exhibited mixed inhibition/induction, particularly inhibiting apoptosis and altering stress and signaling responses.

The tripartite overlap of CDC25C_S216, Rap guanine nucleotide exchange factor 6 (RAPGEF6_S1094), and Zinc finger protein 395 (ZNF395_S449) among positive correlates underscores core hubs in MELK-driven signaling. Phosphorylation of CDC25C at S216, a canonical 14–3-3 binding motif, enforces G2/M checkpoint arrest by cytoplasmic sequestration and inhibition of CDK1 activation-paradoxically, a negative regulatory event that MELK may co-opt to prevent premature mitosis in proliferating cells. This aligns with MELK's established integration into the PLK1-CDC25C-CDK1 axis, where it amplifies mitotic entry while buffering against checkpoint slippage, a mechanism frequently hijacked in cancer to evade DNA damage responses. Similarly, ZNF395_S449 phosphorylation likely enhances its transcriptional activation under hypoxia or stress, promoting interferon-stimulated genes and oncogene expression; given ZNF395′s role as a hypoxia-inducible factor, MELK-mediated modification suggests kinase activity to tumor microenvironment adaptation. RAPGEF6_S1094, a Rap1 guanine nucleotide exchange factor, facilitates T-cell migration and integrin signaling; its positive correlation implies MELK fosters cytoskeletal dynamics essential for invasion, consistent with enrichment of DOCK family (e.g., DOCK5_S1860, DOCK7_S2129) and IQGAP3_S1424 substrates in actin remodeling.

Site-specific patterns further illuminate regulatory diversity. S356, with the largest positive cohort (n = 54), preferentially engages cell cycle effectors like Serine/threonine-protein kinase LATS1 (LATS1_S464) (Hippo pathway)^92^ and MKI67_S374 (proliferation marker),^93^ suggesting a dominant role in unchecked growth-evident in MELK-overexpressing cancers where it sustains Cyclin-dependent kinase 13 (CDK13_S437) mediated splicing for mitotic fidelity. In contrast, S529′s leaner profile (n = 27) emphasizes chromatin modifiers (e.g., SETD2_S2079),^94^ hinting at epigenetic tuning for stemness maintenance, a hallmark of MELK in glioma and breast cancer stem cells. Negative correlates, dominated by Filamin-B (FLNB_S2107) across sites, reveal inhibitory feedback: Filamin B phosphorylation at this actin-binding hinge modulates filopodia formation and receptor trafficking, potentially dampening MELK-induced motility to prevent excessive invasion. Recurrent antagonists like AT-rich interactive domain-containing protein 2 (ARID2_S1300) and TBC1 domain family member 9B (TBC1D9B_S411) (chromatin and Rab-GTPase regulators)^95^ may impose lineage-specific brakes, explaining MELK's dispensability in some normal tissues yet oncogenicity in others.

These findings extend prior phosphoproteomic inferences, where MELK substrates cluster around translation (eIF4B) and NF-κB (SQSTM1) axes in mitosis, but uniquely position site-resolved correlations as a scalable predictor of kinase-substrate pairs. In cancer contexts, where MELK correlates with high mitotic indices and poor prognosis, targeting S356/S505/S529 could disrupt convergent nodes like CDC25C, offering synergy with checkpoint inhibitors. However, correlations do not prove causality; validation via kinase assays (e.g., using recombinant MELK mutants) or CRISPR-edited phosphomimetic models is warranted. Moreover, dataset biases toward tumor-derived phosphoproteomes may overlook tissue-specific substrates, necessitating orthogonal approaches like Kinase Substrate Enrichment Analysis for refinement.

Survival analysis of MELK, MKI67 and TTK

Kaplan-Meier survival analysis of TTK, MELK, and MKI67 expression across BRCA, LUAD, and LIHC cohorts from the TCGA database revealed a consistent and cancer-type-specific prognostic pattern. In LUAD, high expression of all three genes was significantly associated with worse Overall Survival (OS): TTK (HR = 1.7, p = 0.00029), MELK (HR = 1.5, p = 0.0086), and MKI67 (HR = 1.4, p = 0.027). For Disease Free Survival (DFS) in LUAD, only TTK reached significance (HR = 1.5, p = 0.0084), while MELK showed a borderline trend (p = 0.09) and MKI67 was non-significant, suggesting kinase-driven mechanisms more directly influence early recurrence in lung adenocarcinoma.

**Fig. 10:**
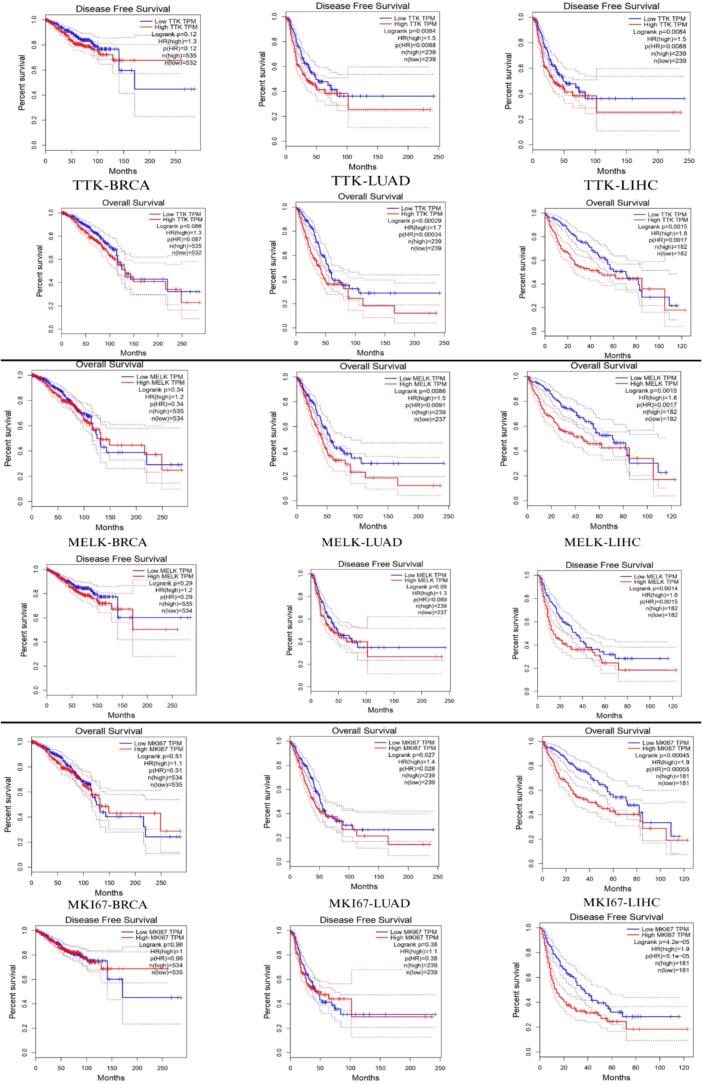
Kaplan-Meier analysis of overall survival (OS) and disease-free survival (DFS) for MELK, MKI67, and TTK in TCGA cohorts.

In LIHC, all three genes demonstrated statistically significant associations across both survival endpoints. MKI67 produced the most powerful finding in the entire analysis, DFS HR = 1.9 with p = 4.2 × 10⁻^5^, followed closely by TTK (OS: HR = 1.8, p = 0.0015) and MELK (OS: HR = 1.8, p = 0.0015; DFS: HR = 1.6, p = 0.0014) (Fig. 10**)**. Kaplan-Meier curves in LIHC showed early and sustained separation, indicating that overexpression of these proliferation-associated genes drives both rapid recurrence and long-term mortality in hepatocellular carcinoma. In contrast, none of the three genes showed statistically significant prognostic associations in BRCA for either OS or DFS, despite the largest sample size among the three cohorts (n ≈ 1,067).

These results demonstrate that TTK, MELK, and MKI67, three functionally related genes involved in cell cycle regulation, mitotic checkpoint control, and tumor proliferation, converge as significant poor prognostic biomarkers in LIHC and LUAD, while showing no prognostic utility in unselected BRCA patients. This cancer-type specificity likely reflects the molecular heterogeneity inherent to breast cancer, where the prognostic impact of proliferation markers is strongly subtype-dependent and is diluted in pan-cohort analyses. The convergence of all three genes as significant prognostic factors in LIHC is particularly compelling. TTK encodes the MPS1 kinase, a master regulator of the spindle assembly checkpoint whose overexpression promotes chromosomal instability and uncontrolled mitotic progression. MELK sustains cancer stem cell self-renewal and G2/M checkpoint bypass, contributing to therapy resistance and early post-treatment tumor regrowth, consistent with the early DFS curve separation observed in LIHC. MKI67, as a universal marker of active cell cycling, reflects the globally elevated proliferative index that underpins the aggressive biology of hepatocellular carcinoma, including vascular invasion and poor differentiation. Together, these findings support the existence of a dysregulated proliferation kinase program in LIHC and LUAD that is clinically meaningful and potentially co-targetable.

## 4 Discussion

The maternal embryonic leucine zipper kinase (MELK) has long been recognized as an oncogenic driver in multiple aggressive cancers,^96^, ^97^ yet the molecular logic governing its activation and downstream signaling has remained elusive. Here, through the largest integrative phosphoproteomics *meta*-analysis of MELK performed to date, we reveal that MELK function is orchestrated by three major regulatory phosphosites, S356, S505, and S529, each operating within distinct yet partially overlapping co-regulatory modules. These sites do not function in isolation but form a sophisticated phosphosite-specific signaling network that integrates mitogenic, mitotic, stress-responsive, and polarity cues, providing a molecular explanation for MELK’s remarkable oncogenic versatility.^97^, ^98^

The most striking observation is the tight positive co-regulation of S356 and S505, evident in both pairwise co-occurrence patterns and shared downstream phosphosites under proliferative conditions. This functional coupling positions S505 as a primary integration hub for diverse upstream kinase families (MAPK, RSK, CaMK, Hippo-related), while S356 appears selectively controlled by mitotic checkpoint kinases NEK4 and TTK/MPS1. Such hierarchical regulation reconciles earlier contradictory reports on MELK essentiality: tumors reliant on rapid mitotic progression may depend predominantly on the S356-NEK4/TTK axis, whereas those driven by sustained mitogenic signaling engage the broader S505 hub. This model offers a plausible mechanism for the observed context-dependent MELK dependency and resistance to pan-MELK inhibitors such as OTSSP167.^99^

A second major insight is the identification of MKI67 (Ki-67) as the universal binary interactor whose phosphorylation state co-varies with all three major MELK sites across virtually all proliferative contexts examined. Given Ki-67′s status as the most widely used clinical proliferation marker, this phosphodynamic linkage provides a mechanistic basis for the strong correlation between MELK expression and tumor mitotic index observed in breast, glioblastoma, and other cancers.^96^, ^97^ Moreover, the consistent co-regulation of CDC25C-S216, a canonical inhibitory phosphorylation that enforces G2/M checkpoint integrity,^100^, ^101^ suggests that MELK-driven tumors may paradoxically maintain elevated checkpoint signaling to prevent catastrophic mitosis while still accelerating cell cycle progression, a hallmark of oncogene-induced replication stress.^102^

The site-specific phosphatase and substrate networks further underscore functional segregation. Whereas S356 and S505 predominantly drive pro-proliferative and pro-survival programs through positive regulation of cell cycle effectors (CDC25C, LATS1, MKI67) and chromatin organizers (CBX5, EZH2), S529 exhibits mixed inductive/inhibitory effects enriched in stress adaptation, apoptosis inhibition, and cytoskeletal polarity (CAMK2G, RAF1, ROCK1). This tripartite organization mirrors the broader theme emerging from kinome-wide phosphoproteomics studies: individual phosphosites on the same kinase can decode distinct input signals and execute non-redundant outputs, dramatically expanding signaling capacity beyond simple on/off switching.^103^

Our phosphosite-specific co-regulation networks offer a molecular framework to contextualize and potentially unify prior mechanistic observations. The tightly coupled S356/S505 proliferative–mitotic module, driven by convergent MAPK/RSK/CaMK/Hippo and spindle-checkpoint (NEK4/TTK) inputs, aligns with MELK’s documented roles in cell-cycle progression, AKT/mTOR signaling, and tumorigenesis. For example, xCT-mediated upregulation of MELK activates AKT/mTOR to promote colorectal cancer growth^104^ and stemness, processes that our data link to S505-enriched pathways (DNA replication, cell-cycle progression, and cancer-associated signaling). Similarly, tumor cell-intrinsic MELK drives CCL2-dependent immunosuppression and radiotherapy resistance in HCC, consistent with the mitogenic and chromatin-regulatory outputs of the S356/S505 axis.^105^ In contrast, the partially antagonistic S529 module, enriched in stress, calcium, metabolic, and cytoskeletal polarity signals, resonates with MELK’s functions in inflammation and adaptive responses. Pharmacological MELK inhibition limits ferroptosis and inflammatory signaling in colitis models and reduces colitis-propelled carcinogenesis, suggesting that S529 may integrate stress cues that sustain pro-tumorigenic inflammation or therapy resistance.^106^

The universal MELK-MKI67 phosphosite coupling suggests that phospho-MELK or its co-regulated partners could serve as more dynamic proliferation biomarkers than total protein levels. The selective engagement of mitotic kinases at S356 raises the possibility that combination therapies targeting both MELK and spindle assembly checkpoint components (e.g., TTK inhibitors currently in clinical trials) could achieve synthetic lethality in MELK-dependent tumors. Most importantly, the pronounced modularity revealed here argues strongly for the development of phosphosite-selective MELK modulators an ambitious but increasingly feasible goal given recent advances in covalent and allosteric kinase inhibition strategies.

In conclusion, by moving beyond total MELK abundance to phosphosite-resolved co-regulation networks, we provide the first comprehensive regulatory atlas of this enigmatic kinase. MELK emerges not as a monolithic oncogenic driver but as a finely tuned signaling node capable of context-specific responses through three major phosphosites that integrate mitogenic, mitotic, and stress inputs. These findings illuminate its role in proliferative and adaptive oncogenic states, and nominate precise intervention points for the next generation of anti-MELK therapies in hyperproliferative malignancies.

## 5 Limitations and future perspectives

Our co-regulation approach, while statistically rigorous and multi-evidence filtered, relies on correlative patterns across heterogeneous datasets and cannot formally prove direct kinase–substrate relationships or causal regulatory mechanisms. Correlations observed between MELK phosphosites (S356, S505, S529) and co-regulated PsOPs, upstream kinases, phosphatases, binary interactors, or downstream substrates may reflect indirect effects, shared upstream regulators, or parallel pathway activation rather than direct phosphorylation by or of MELK. Furthermore, the majority of underlying studies were performed in cancer cell lines, potentially biasing toward hyperproliferative states and underrepresenting physiological regulation. Finally, although S356, S505, and S529 clearly dominate global datasets, rare or context-specific sites may have been overlooked.

While this phosphosite-resolved regulatory atlas of MELK provides a robust foundation for hypothesis testing, direct causality remains to be established through targeted validation. Future studies could include: (1) site-directed mutagenesis of S356, S505, and S529 in MELK-knockout/knockdown cancer cells to evaluate effects on mitotic progression, proliferation, chromatin architecture, stress responses, and interactions with MKI67/CDC25C; (2) phospho-specific antibodies against pS356, pS505, and pS529 to track site-specific dynamics across cell-cycle phases or upon MAPK, NEK4/TTK, or CaMK pathway perturbations; (3) in vitro kinase assays using recombinant wild-type versus mutant MELK with substrates such as CDC25C_S216, MKI67 phosphosites, or EZH2_T487 to verify direct phosphorylation; and (4) functional assays with kinase-dead MELK mutants or selective inhibitors, coupled with phosphosite readouts, to delineate module-specific contributions (S356/S505 mitotic-proliferative axis vs. S529 stress-polarity module) to oncogenic traits like therapy resistance and invasion. Such experiments will confirm mechanistic links and refine the proposed modular MELK signaling model.

Looking ahead, combining our findings with new single-cell and spatial phosphoproteomics tools offers exciting potential. Bulk data averages signals from mixed cell types, hiding differences tied to cell-cycle stages, cell types, or local environments that control the three MELK modules. Single-cell phosphoproteomics, paired with cell-cycle syncing or stress tests, could map how S356, S505, and S529 phosphorylation changes in individual cells (proliferating, resting, or stressed). This would sharpen our model of MELK signaling in specific contexts.

## 6 Conclusions

This study establishes the first phosphosite-specific regulatory atlas of maternal embryonic leucine zipper kinase (MELK) by integrative analysis of over 3,800 global human phosphoproteomics datasets. We demonstrate that MELK signaling is not governed by uniform phosphorylation but by three major regulatory sites, S356, S505, and S529, that operate as functionally distinct yet interconnected modules. S356 and S505 form a tightly coupled mitotic–proliferative axis under convergent control by MAPK, RSK, CaMK, Hippo, and spindle-checkpoint kinases (NEK4, TTK/MPS1), whereas S529 serves as a stress- and polarity-responsive sensor with partially antagonistic outputs. MKI67 (Ki-67) emerges as the universal phosphodynamic partner across all three sites, mechanistically linking MELK activity to cellular proliferation, while CDC25C-S216 and a broad network of phosphatases, substrates, and binary interactors reveal how MELK simultaneously accelerates cell cycle progression and maintains adaptive checkpoints. Importantly, survival analysis across TCGA cohorts demonstrates that high MELK and MKI67 expression, together with the co-regulated mitotic kinase TTK, correlates with poor prognosis in lung adenocarcinoma and hepatocellular carcinoma, thereby translating the identified phosphosite-resolved proliferative modules into clinically relevant prognostic signatures.

These findings sheds light into the longstanding paradox of MELK’s context-dependent essentiality in cancer, explain its frequent correlation with poor prognosis despite debated “non-essentiality” in some models, and provide a potential molecular framework for its oncogenic versatility. Clinically, the phosphosite-resolved map nominates dynamic phospho-biomarkers for tumor proliferation, highlights synthetic-lethal opportunities with mitotic checkpoint inhibitors, and strongly motivates the development of site-selective or module-specific MELK therapeutics.

## Funding

This research did not receive any specific grant from funding agencies in the public, commercial, or not-for-profit sectors.

## CRediT authorship contribution statement

**Noreen A Khan:** Writing – original draft, Investigation, Formal analysis. **Amal Fahma:** Supervision, Investigation. **Althaf Mahin:** Resources, Methodology, Data curation. **Athira Perunelly Gopalakrishnan:** Resources, Methodology, Investigation, Data curation. **Prathik Basthikoppa Shivamurthy:** Software, Resources. **Samseera Ummar:** Visualization, Software. **Athira C. Rajeev:** Writing – review & editing, Supervision. **Rajesh Raju:** Writing – review & editing, Project administration, Funding acquisition, Conceptualization.

## Declaration of competing interest

The authors declare that they have no known competing financial interests or personal relationships that could have appeared to influence the work reported in this paper.
